# Health literacy of Dutch adults: a cross sectional survey

**DOI:** 10.1186/1471-2458-13-179

**Published:** 2013-02-27

**Authors:** Iris van der Heide, Jany Rademakers, Maarten Schipper, Mariël Droomers, Kristine Sørensen, Ellen Uiters

**Affiliations:** 1 Centre for Nutrition, Prevention and health Services, National Institute for Public Health and the Environment, Bilthoven, Netherlands; 2 NIVEL, Netherlands Institute for Health Services Research, Utrecht, Netherlands; 3 Department of Statistics, Mathematical Modelling and Data Logistics, National Institute for Public Health and the Environment, Bilthoven, Netherlands; 4 Department of Public Health, Academic Medical Center, University of Amsterdam, Amsterdam, Netherlands; 5 Department of International Health, Research School of Primary Care and Public Health, Maastricht University, Maastricht, Netherlands

**Keywords:** Health literacy, Socio-economic position, General population, Netherlands

## Abstract

**Background:**

Relatively little knowledge is available to date about health literacy among the general population in Europe. It is important to gain insights into health literacy competences among the general population, as this might contribute to more effective health promotion and help clarify socio-economic disparities in health. This paper is part of the European Health Literacy Survey (HLS-EU). It aims to add to the body of theoretical knowledge about health literacy by measuring perceived difficulties with health information in various domains of health, looking at a number of competences. The definition and measure of health literacy is still topic of debate and hardly any instruments are available that are applicable for the general population. The objectives were to obtain an initial measure of health literacy in a sample of the general population in the Netherlands and to relate this measure to education, income, perceived social status, age, and sex.

**Methods:**

The HLS-EU questionnaire was administered face-to-face in a sample of 925 Dutch adults, during July 2011. Perceived difficulties with the health literacy competences for accessing, understanding, appraising and applying information were measured within the domains of healthcare, disease prevention and health promotion. Multiple linear regression analyses were applied to explore the associations between health literacy competences and education, income, perceived social status, age, and sex.

**Results:**

Perceived difficulties with health information and their association with demographic and socio-economic variables vary according to the competence and health domain addressed. Having a low level of education or a low perceived social status or being male were consistently found to be significantly related to relatively low health literacy scores, mainly for accessing and understanding health information.

**Conclusions:**

Perceived difficulties with health information vary between competences and domains of health. Health literacy competences are associated with indicators of socio-economic position and with the domain in which health information is provided.

## Background

During the past decades, there has been a growing interest in the concept of health literacy, accompanied by the increased emphasis on the role and responsibility of citizens in health and healthcare [1-3]. The importance of health literacy as a topic of research has been pointed out by a number of studies that suggest health literacy might play a significant role in maintaining or improving health and could be an unexplored predictor of health disparities [4-6]. The aim of the present study is to contribute to the theoretical knowledge being built up about health literacy. An initial insight into the health literacy of the general population will therefore be provided and its association with demographic and socio-economic characteristics will be examined.

An important issue regarding health literacy research is the ongoing debate on the definition and scope of health literacy [1,7,8]. Within this discourse, two main approaches can be distinguished, namely the ‘clinical’ approach and the ‘public health’ approach [7,9]. Key elements of definitions from a clinical perspective include elements of behaviour that mainly reflect individual competences needed to function in the role of patient within a healthcare environment [8,10,11]. Studies that define health literacy from a public health point of view extend the concept by addressing the public at large instead of patients and including dimensions beyond the medical context, such as the workplace, political arena or home [4,12]. Most research on health literacy stems from the clinical perspective. Additionally, research that stems from the public health perspective has mainly been carried out outside Europe, although there has been some recently in Switzerland [4,13-15]. Especially in the European situation, little is known about health literacy outside a clinical setting and among the general population.

The present paper is based on data gathered in the Netherlands in the context of the European Health Literacy Survey (HLS-EU). A research consortium with members from Austria, Bulgaria, Germany, Greece, Ireland, the Netherlands, Poland and Spain made joint efforts to develop a common tool to measure health literacy in the general population [16,17]. This tool was based on a common definition and conceptual model of health literacy (see Additional file 1). Other measurement tools often measured health literacy as a unidimensional concept by focusing on text comprehension or word recognition [18-20]. The HLS-EU addresses health literacy as a multidimensional concept, embracing competences other than reading skills. More precisely, health literacy is measured as people’s perceived ability to access, understand, appraise, and apply health information. This measure is not limited to a single domain, but involves three domains of health, namely healthcare, disease prevention and health promotion, which have been clarified in more detail elsewhere [17].

In addition to the development of an instrument (HLS-E-Q), the HLS-EU survey gathered information on socio-economic and demographic characteristics. Previous studies suggest that low health literacy is more prevalent among people with low levels of education and low incomes and those who are older [5,6,21-24]. As far as is known, perceived social status, an increasingly-used measure for socio-economic status, has not been studied in relation to health literacy before, but was included in this study as well [25]. As regards health literacy of men and women, the studies report varying results. Whereas some found no relationship [4,25], others report that one of the sexes is more likely to have low health literacy [26,27].

Better insights into the relationships between demographic and socio-economic characteristics and health literacy will help identify vulnerable groups with limited health literacy who are therefore possibly at risk of being in poor health [28]. Specific questions in our study therefore are 1) to what extent do adults perceive difficulties with the health literacy competences of accessing, understanding, appraising and applying health information in the domains of healthcare, disease prevention and health promotion and 2) to what extent are these competences related to demographic and socio-economic characteristics?

## Methods

### Study design and data collection

A stratified random sampling design was applied to the Dutch population aged 15 years or older, in accordance with the Eurobarometer methodology [29]. The sample was stratified according to province and within provinces according to urban and rural areas, leading to a probability of inclusion that was proportional to population size and density. In the first stage, 699 areas were defined, including a total selection of 24,942 addresses. Households were selected randomly in each of these areas. In each household, one respondent was recruited over the phone or contacted by e-mail, following the closest birthday rule. During July 2011, 221 interviewers administered the questionnaires face-to-face with the pre-recruited respondents in people’s homes. The questionnaires were conducted in Dutch. A total of 2817 people were contacted of whom 1794 were not willing to participate, leading to a sample of 1023 participants (response rate 36%). It was decided to focus the analyses on persons aged 25 years or older, as income, education and social status are more stable after this age, resulting in a final sample of 925 adults.

### Assessment of variables

#### Health literacy

The questionnaire for measuring health literacy in the general population in Europe (the HLS-EU-Q) was developed collectively by the HLS-EU consortium [16]. The questionnaire was based on the definition and conceptual model developed within the HLS-EU consortium, which are presented in an Additional file 1 and described in detail elsewhere [17]. Table 1 presents four examples of items from the HLS-EU-Q. For the full questionnaire, please contact the fifth author (KS). The HLS-EU-Q was pre-tested for understandability and completeness, using focus groups in Greece, Ireland and the Netherlands (n = 6 in each country) and face-to-face interviews in Ireland and the Netherlands (n = 50 in each country). Extensive information on the development and pre-testing of the HLS-EU-Q is described elsewhere [17,30]. The final questionnaire, as illustrated in Table 2, measured health literacy across three domains of health, namely healthcare, disease prevention and health promotion. Within these domains, the questionnaire focused on (a) accessing, or the ability to seek, find and obtain health information (13 items); (b) understanding, or the ability to comprehend health information (11 items); (c) appraising, or the ability to interpret, filter, judge and evaluate health information (12 items); and (d) applying, or the ability to communicate and use the information to maintain and improve health (11 items). Answer categories on the health literacy questions (all phrased similarly to “On a scale from very easy to very difficult, how easy would you say it is to understand why you need health screenings?”) were on a 4-point Likert-type scale ranging from 1 = very difficult, 2 = fairly difficult to 3 = fairly easy and 4 = very easy. A “don’t know” answer option was not provided, but only used when stated spontaneously. This response was coded as a missing value. In order to get an initial insight into perceived difficulties, answers were combined to sum scores per competence and a distinction was made between those who perceive numerous difficulties and those who perceive few difficulties. Those respondents with the lowest scores on all four competences (scores below the first quartile for accessing, understanding, appraising as well as applying) were categorized as perceiving numerous difficulties; those with the highest scores were considered to perceive few difficulties (scores above the third quartile for accessing, understanding, appraising as well as applying). This was done per domain as well as over all domains.

**Table 1:** Examples of HLS-EU questionnaire items per competence and domain

**Competence / health domain**	**On a scale from very easy to very difficult, how easy would** **you say it is for you to…**
accessing / healthcare	…find out what to do in case of a medical emergency?
understanding / healthcare	…understand what your doctor says to you?
appraising / disease prevention	…judge which vaccinations you may need?
applying / health promotion	…make decisions to improve your health?

**Table 2:** Competences and domains included in the HLS-EU questionnaire

**Competences**	**Domains**		
	***Healthcare***	***Disease prevention***	***Health promotion***
*Accessing*	The ability to seek, find and obtain information on medical or clinical issues.	The ability to seek, find and obtain information on risk factors for health.	The ability to seek, find and obtain information on determinants of health.
*Understanding*	The ability to comprehend information on medical or clinical issues.	The ability to comprehend information on risk factors and derive meaning.	The ability to comprehend information on determinants of health and derive meaning.
*Appraising*	The ability to interpret, filter, judge, and evaluate information on medical or clinical issues.	The ability to interpret, filter, judge, and evaluate information on risk factors.	The ability to interpret, filter, judge, and evaluate information on determinants of health.
*Applying*	The ability to communicate and use information on medical or clinical issues to make informed decision.	The ability to communicate and use information on risk factors to make informed decisions.	The ability to communicate and use information on determinants of health to make informed decisions.

#### Demographic and socio-economic characteristics

The following demographic and socio-economic characteristics were analysed: sex, age, educational level, net household income per month and social status (see Table 3). Age was measured and analysed as a continuous variable. Educational level was measured on a six-point scale. For the purpose of the analyses, education was categorized into 1) no education or primary education, 2) lower secondary education, 3) (upper) secondary education or post-secondary non-tertiary education (including vocational education), or 4) tertiary education (bachelor’s degree or higher). Net monthly household income was measured on a ten-point scale. In the analyses, income was recoded into quartiles 1) less than 1850 euros, 2) 1850 – 2400 euros, 3) 2400 – 3600 euros, 4) 3600 euros or more. As regards perceived social status: there is evidence that this self-reported variable reflects standard markers of socio-economic status such as education and income, as well as having the advantage of being seen as a more accurate measure of social position [26]. In this study, perceived social status was assessed by the answer to the question: “On the following scale, step 1 corresponds to ‘the lowest level in society’; step 10 corresponds to ‘the highest level in society’. Could you tell me which step you would say you were on?” This item stemmed from the Eurobarometer [31]. Table 3 presents the scores in the categories low (1-4), medium (5, 6) and high (7-10), continuous scores were included in the analyses.

**Table 3:** Characteristics study participants

**Characteristics**	**Total (n = 925)**		**Men (n = 439)**		**Women (n = 486)**	
	**%**	**n**	**%**	**n**	**%**	**n**
Age in categories						
25 – 34	13.1	(121)	14.4	(63)	11.9	(58)
35 – 44	14.5	(134)	13.9	(61)	15.0	(73)
45 – 54	18.7	(173)	16.9	(74)	20.4	(99)
55 – 64	16.0	(148)	15.3	(67)	16.7	(81)
65 – 74	19.4	(179)	21.2	(93)	17.7	(86)
75 – 84	15.1	(140)	15.3	(67)	15.0	(73)
85+	3.2	(30)	3.2	(14)	3.3	(16)
Missing values	0	(0)	0	(0)	0	(0)
Highest completed education level						
No education or primary education	6.9	(64)	5.9	(26)	7.8	(38)
Lower secondary education	26.2	(242)	26.0	(115)	26.1	(127)
(Upper or post-) secondary non-tertiary education	29.1	(269)	29.0	(127)	29.2	(142)
Tertiary education (bachelor’s degree or higher)	37.8	(350)	39.0	(171)	36.8	(179)
Missing values	1.0	(9)	0.7	(3)	1.2	(6)
Household net income per month in euros						
< 1850	35.3	(326)	29.6	(130)	40.3	(196)
1850 – 2400	16.8	(155)	17.1	(75)	16.5	(80)
2400 – 3600	29.5	(273)	32.1	(141)	27.2	(132)
3600 or >	18.5	(171)	21.2	(93)	16.0	(78)
Missing values	16.2	(150)	16.4	(72)	16.0	(78)
Perceived social status						
Low	3.7	(35)	3.6	(16)	3.8	(19)
Medium	24.2	(224)	22.8	(100)	25.5	(124)
High	72.0	(666)	73.6	(323)	70.6	(343)
Missing values	2.7	(25)	3.4	(15)	2.1	(10)

### Statistical analyses

To explore reliability and internal consistency of the questionnaire, factor analyses (principal component analyses with promax rotation: data not shown) were performed and Cronbach’s alphas were calculated. The internal consistency of the health literacy items (measuring accessing, understanding, appraising and applying) was satisfactory (Cronbach’s α = 0.84, 0.83, 0.85 and 0.78 respectively). In accordance with the domains defined beforehand, factor analyses distinguished between the factors ‘healthcare’, ‘disease prevention’ and ‘health promotion’ for the competences ‘accessing’, ‘understanding’ and ‘applying’. With regard to appraising, the factor analysis identified only two factors, which were labelled ‘healthcare and disease prevention’ and ‘health promotion’, combining the items designed to measure health literacy in healthcare and disease prevention. The reliability and internal consistency analyses justified the calculation of sum scores for each combination of competence and domain. Descriptive statistics in terms of sum scores and means per item (sum score / number of items) were performed to answer the first research question. Multiple linear regression analyses were used to answer the second research question concerning the associations between health literacy and demographic and socio-economic characteristics. For each competence within each domain, a multiple linear regression analysis was performed in SAS 9.2, including the means per item for each health literacy competence, education, income, social status, age, and sex.

#### Missing values

The dataset contained missing values concerning education (1%, N = 9), subjective social status (3%, N = 25) and income (16%, N = 150), as presented in Table 3. Table 4 shows the missing values per health literacy domain and competence. The characteristics of the missing values on the health literacy competences are described in Additional file 2. The method of multiple imputations by chained equations was used to handle the presence of missing values in the study data, as these were not missing completely at random (see Additional file 2 for more details on the missing data) [32]. Following this procedure, the original data set was completed 20 times. Each imputed dataset was analysed separately and the outcomes of each analysis were combined according to Rubin’s rules to obtain the outcome of the whole analysis, which incorporates the uncertainty due to the missing values [31]. The imputations were done in R 2.14.0, with use of the mice package [31,32]. Recent studies have shown that this technique provides less biased results compared to a complete case analysis and is considered to be the state-of-the-art method for dealing with missing data [33,34].

**Table 4:** **Descriptive statistics per health literacy competence and domain (N = 925)**^**a**^

**Competence domain**	**Items**	**Missings (n)**^**b**^	**Mean (SD)**	**Mean per item (SD)**	**Mean per item per quartile**		
					**25**	**50**	**75**
Accessing	11	231	35.2 (5.2)	3.2 (0.5)	2.6	3.2	3.7
Healthcare	3	82	9.7 (1.8)	3.2 (0.6)	2.7	3.3	3.4
Disease prevention	6	122	20.3 (3.1)	3.4 (0.5)	2.8	3.4	4.0
Health promotion	2	118	5.1 (1.7)	2.6 (0.8)	1.7	2.5	3.9
Understanding	11	157	36.8 (4.9)	3.3 (0.5)	2.8	3.4	3.9
Healthcare	4	58	13.5 (2.1)	3.4 (0.5)	2.8	3.4	3.9
Disease prevention	3	29	10.8 (1.5)	3.6 (0.5)	3.0	3.7	4.0
Health promotion	4	108	12.5 (2.6)	3.1 (0.7)	2.4	3.2	3.9
Appraising	12	219	36.7 (5.9)	3.1 (0.5)	2.5	3.1	3.7
Healthcare and prevention	9	201	27.0 (4.8)	3.0 (0.5)	2.4	3.0	3.6
Health promotion	3	47	9.8 (1.9)	3.3 (0.6)	2.7	3.5	4.0
Applying	9	196	28.9 (4.2)	3.2 (0.5)	2.7	3.2	3.8
Healthcare	3	16	10.8 (1.5)	3.6 (0.5)	3.0	3.7	4.0
Disease prevention	2	64	5.7 (1.7)	2.9 (0.8)	1.8	2.8	3.8
Health promotion	4	155	12.4 (2.7)	3.1 (0.7)	2.3	3.1	3.8

^a ^Scores ranged from ‘very difficult’ (lowest score) to ‘very easy’ (highest score).

^b ^Number of missing values per competence and domain before multiple imputation.

## Results

The sample distribution in terms of sex, education and income (Table 3) was in accordance with the distribution in the Dutch population (not tabulated) [35]. Adults aged 65 years or older were overrepresented and adults between 25 and 39 years of age were underrepresented in the studied population [35].

Concerning the four competences of accessing, understanding, appraising and applying health information, the mean scores per item are all close to 3 (equal to being perceived as easy) where the maximum score is 4 (being perceived as very easy) (see Table 4). The mean score per item (over all domains) is lowest for appraising information (3.1) and highest for understanding information (3.4). The scores presented in Table 4 imply that perceived difficulties vary between the health domains. Accessing health information seems to be perceived as more difficult in the domain of health promotion (mean score per item is 2.6) than in the domain of disease prevention (mean score per item is 3.4).

Comparing the health domains of healthcare, disease prevention and health promotion, the mean score per item (over all competences) was lowest in the domain of health promotion (3.0) and highest in the domain of healthcare (3.3) (not tabulated). Within the domain of healthcare, 9.6% of Dutch adults (N = 89) perceived numerous difficulties, whereas 14.4% (N = 134) perceived few difficulties (not tabulated). Furthermore, 4.8% (N = 44) perceived numerous difficulties with information on disease prevention and 12.0% (N = 111) perceived few difficulties (not tabulated). Finally, 9.2% (N = 86) perceived numerous difficulties with information on health promotion, whereas 8.6% (N = 79) perceived few difficulties (not tabulated). Looking at the respondents who perceived numerous difficulties across all three health domains, it appeared that this was the case for 10.4% (N = 96) of the respondents (those scoring below the first quartile on accessing, understanding, appraising as well as applying). Subsequently, 11.9% (N = 110) of the respondents perceived few difficulties (scores above the third quartile on accessing, understanding, appraising as well as applying) across all domains (not tabulated).

As regards the associations of socio-economic and demographic variables with health literacy, none of the included socio-economic and demographic variables was consistently associated with all health literacy competences and domains addressed. Overall, it was found that people with a lower level of education, or who reported a lower perceived social status or were male perceived more difficulties with health literacy. Taking into account all demographic and socio-economic variables, the results of the multiple regression analyses indicate a clear association of health literacy with level of education (see Table 5). The group with the lowest educational level in particular had significantly lower health literacy scores compared to the group with the highest educational level, indicating that the former group experienced more difficulties. However, this association differed between the competences addressed and was most obvious for the competences of accessing and understanding health information in the domains of healthcare and disease prevention. In the domain of health promotion the association was most obvious for understanding health information. With regard to accessing and understanding, the lowest income group was also found to have lower health literacy scores compared to those with the highest incomes. However, this was only found in the healthcare domain (Table 5). In addition to the socio-economic indicators of education and income, social status was also found to be related to health literacy. The higher the self-reported social status, the higher the health literacy scores, except for accessing information on healthcare and health promotion and applying information on disease prevention. Age was found to be significantly associated with health literacy scores in some domains. Like education, age was mainly significantly related to accessing and understanding health information, but also to some extent to appraising health information in the domain of health promotion. In terms of sex, men perceived more difficulties than women, except for accessing and applying in the domain of health promotion.

**Table 5:** Associations between socio-economic and demographic characteristics and health literacy competences *

***Healthcare***	**Accessing**		**Understanding**		**Appraising**^**a**^		**Applying**	
	**B**	**95% CI**	**B**	**95% CI**	**B**	**95% CI**	**B**	**95% CI**
Education (reference group: tertiary education)								
No education or primary education	**−0.2906**	**(-0.4653 to -0.1160)**	**−0.1949**	**(-0.3497 to -0.0402)**	−0.0562	(-0.2098 to 0.0974)	**−0.1576**	**(-0.3021 to -0.0131)**
Lower secondary education	−0.0464	(-0.1558 to 0.0631)	**−0.1838**	**(-0.2772 to -0.0904)**	−0.0074	(-0.1030 to 0.0882)	−0.0678	(-0.1556 to 0.0200)
(Upper or post-) secondary education ^b^	0.0557	(-0.0430 to 0.1544)	−0.0587	(-0.1432 to 0.0256)	0.0502	(-0.0368 to 0.1371)	−0.0431	(-0.1229 to 0.0367)
Income (reference group: 3600 or more)								
< 1850 euros	**−0.2294**	**(-0.3738 to -0.0849**)	**−0.1283**	**(-0.2444 to -0.0121)**	−0.0418	(-0.1593 to 0.0758)	0.0051	(-0.1082 to 0.1183)
1850 – 2400 euros	-0.0771	(-0.2304 to 0.0762)	0.0065	(-0.1192 to 0.1322)	0.0267	(-0.1000 to 0.1535)	0.0748	(-0.0432 to 0.1927)
2400 – 3600 euros	**-0.1426**	**(-0.2610 to -0.0156)**	-0.0510	(-0.1562 to 0.0542)	-0.0554	(-0.1668 to 0.0561)	-0.0122	(-0.1187 to 0.0944)
Social status	**0.0258**	**( 0.0067 to 0.0583)**	**0.0371**	**( 0.0087 to 0.0654)**	**0.0584**	**( 0.0295 to 0.0874)**	**0.0308**	**( 0.0036 to 0.0581)**
Age (years)	**-0.0039**	**(-0.0062 to -0.0016)**	-0.0006	(-0.0025 to 0.0014)	0.0010	(-0.0010 to 0.0031)	-0.0002	(-0.0021 to 0.0017)
Male	**0.0810**	**( 0.0037 to 0.1582)**	**0.0799**	**( 0.0143 to 0.1455)**	**0.1415**	**( 0.0734 to 0.2096)**	**0.0673**	**( 0.0048 to 0.1298)**
*Disease prevention*	**Accessing**		**Understanding**				**Applying**	
	B	95% CI	B	95% CI			B	95% CI
Education (reference group: tertiary education)								
No education or primary education	**−0.2418**	**(-0.3970 to -0.0866)**	**−0.2069**	**(-0.3489 to -0.0650)**			−0.1217	(-0.3687 to 0.1253)
Lower secondary education	**−0.0989**	**(-0.1897 to -0.0081)**	**−0.1276**	**(-0.2137 to -0.0415)**			−0.1028	(-0.2530 to 0.0473)
(Upper or post-) secondary education ^b^	0.0057	(-0.0772 to 0.0887)	−0.0152	(-0.0945 to 0.0640)			−0.0426	(-0.1807 to 0.0955)
Income (reference group: 3600 or more)								
< 1850 euros	−0.1025	(-0.2175 to 0.0125)	−0.0516	(-0.1671 to 0.0640)			0.0098	(-0.1840 to 0.2037)
1850 – 2400 euros	0.0506	(-0.0775 to 0.1787)	0.0283	(-0.0927 to 0.1493)			0.0402	(-0.1644 to 0.2447)
2400 – 3600 euros	−0.0236	(-0.1288 to 0.0816)	−0.0138	(-0.1182 to 0.0906)			−0.0137	(-0.1995 to 0.1721)
Social status	**0.0485**	**( 0.0208 to 0.0763)**	**0.0313**	**( 0.0050 to 0.0577)**			0.0242	(-0.0215 to 0.0699)
Age (years)	**−0.0028**	**(-0.0047 to -0.0008)**	**−0.0020**	**(-0.0039 to -0.0001)**			−0.0011	(-0.0043 to 0.0022)
Male	**0.1498**	**( 0.0849 to 0.2148)**	**0.0839**	**( 0.0221 to 0.1457)**			**0.1501**	**( 0.0427 to 0.2575)**
*Health promotion*	**Accessing**		**Understanding**		**Appraising**		**Applying**	
	B	95% CI	B	95% CI	B	95% CI	B	95% CI
Education (reference group: tertiary education)								
No education or primary education	−0.0779	(-0.3309 to 0.1751)	**−0.3552**	**(-0.5395 to -0.1709)**	−0.0881	(-0.2678 to 0.0916)	−0.1797	(-0.3774 to 0.0181)
Lower secondary education	−0.0266	(-0.1798 to 0.1266)	**−0.2267**	**(-0.3390 to -0.1143)**	−0.0538	(-0.1642 to 0.0565)	−0.0170	(-0.1350 to 0.1009)
(Upper or post-) secondary education ^b^	0.0827	(-0.0578 to 0.2232)	−0.1013	(-0.2057 to 0.0031)	0.0752	(-0.0259 to 0.1762)	−0.0037	(-0.1122 to 0.1049)
Income (reference group: 3600 or more)								
< 1850 euros	−0.1036	(-0.2928 to 0.0857)	−0.0296	(-0.1669 to 0.1077)	0.0510	(-0.0919 to 0.1938)	−0.0146	(-0.1656 to 0.1364)
1850 – 2400 euros	0.0513	(-0.1546 to 0.2572)	0.0544	(-0.1003 to 0.2090)	0.1318	(-0.0226 to 0.2862)	0.0683	(-0.0928 to 0.2293)
2400 – 3600 euros	−0.0363	(-0.2068 to 0.1342)	−0.0029	(-0.1331 to 0.1273)	0.0048	(-0.1266 to 0.1362)	−0.0079	(-0.1510 to 0.1351)
Social status	0.0348	(-0.0120 to 0.0815)	**0.0543**	**(0.0200 to 0.0886)**	**0.0686**	**(0.0346 to 0.1027)**	**0.0629**	**(0.0264 to 0.0994)**
Age (years)	**0.0052**	**(0.0018 to 0.0085)**	**−0.0028**	**(-0.0053 to -0.0004)**	**0.0063**	**(0.0039 to 0.0087)**	0.0006	(-0.0020 to 0.0032)
Male	−0.0093	(-0.1183 to 0.0996)	**0.1975**	**(0.1168 to 0.2782)**	**0.1445**	**(0.0651 to 0.2239)**	0.0458	(-0.0398 to 0.1314)

* Analyses are based on mean per item; significant differences are printed in bold (p < 0.05). ^a ^Scores for appraising health information in the domain of healthcare and disease prevention were combined based on outcomes of factor analysis. ^b ^Non-tertiary including vocational education.

## Discussion

The aim of this study was to contribute to theoretical knowledge being built up about health literacy and to provide an initial insight into the health literacy of the general population and its associations with demographic and socio-economic characteristics. For that purpose, the Dutch data from the European Health Literacy Survey (HLS-EU) was used [16,17]. The data provided information on health literacy reflected by the competences of accessing, understanding, appraising and applying information in the domains of healthcare, disease prevention and health promotion. The findings of this study suggest that the health literacy scores in a sample of the Dutch general population vary between the four different competences studied. This is consistent with findings from other countries participating in the HLS-EU project [16]. Furthermore, the perceived difficulties with these competences differ according to the health domain which they appeal to. For example, accessing information on healthcare was perceived more difficult than accessing information on disease prevention. It seems that the level of health literacy in the general population is associated with the setting in which the health information is provided.

In terms of health outcomes, low health literacy scores in the three domains and over the four competences might lead to suboptimal health in various ways. For example, those who perceive difficulties with accessing and understanding information about screening might unintentionally be excluded from screening programmes [36,37]. Moreover, those who perceive difficulties understanding their medication leaflets might not be able to use their medication correctly [38].

As to the extent to which health literacy competences are associated with demographic and socio-economic characteristics, the results indicate that lower health literacy is associated with lower socio-economic position, in accordance with the literature [4-6,21-23]. Educational and income-related differences in health literacy were found in particular in accessing and understanding health information. These can be considered as basic health literacy competences, related to functional health literacy as described by Nutbeam [7]. Differences between educational levels or income groups were found to a lesser extent for appraising and applying health information, which are more complex competences related to the concept of critical health literacy [7]. This indicates that those who are highly educated or have a high income do not have an advantage on the more complex competences of appraising and applying compared to those who are low educated or have a low income, as is the case for the basic competences of accessing and understanding. It is recommended that these aspects and the aspect of critical health literacy should be addressed in future research.

One striking finding is that perceived social status seems to affect all health literacy competences. This leads to the assumption that this subjective indicator of socio-economic status differs, in relation to health literacy, from the objective indicators of education and income. Given the importance that has been placed on the association between health literacy and socio-economic status, this seems an important topic for future research [6,7].

This study found mixed results regarding the association between health literacy and age. A negative association was found between age and accessing information on healthcare and disease prevention and understanding information on disease prevention and health promotion, indicating that increasing age is accompanied by lower levels of health literacy on these specific dimensions. This seems in accordance with findings of former studies [4,6,21,22,24]. It has been suggested that the negative association between age and health literacy might be attributable to an age related decline of the ability to perform cognitive tasks that require information processing [39]. Older adults seem to have more difficulty completing tasks that require reasoning or inferences from information presented to them, which has been linked to lower health literacy [39]. However, for accessing and appraising health information on health promotion, the present study suggest a positive association between age and health literacy, which seems contradicting to what was found in other studies. Further research is needed to examine why the direction of the association between age and health literacy differs between health literacy dimensions. The fact that elderly in the Netherlands are relatively high educated could be a possible contributor to the finding of mixed results.

With regard to the association between health literacy and sex, it was found that females perceive fewer difficulties with health information than men, especially in the domains of healthcare and disease prevention. No consistent pattern between sex and health literacy has been reported in the literature [4,6,26,27].

The results of our study suggest that health information, whether it is about healthcare, disease prevention or health promotion, will most likely not have the same effect across the various socio-economic strata in the Dutch population. This implies that there could be benefits from improving the accessibility and usability of health information. In addition, a more challenging task seems to lie in making information easier to judge and to apply, as this calls upon more complex cognitive capacities [38]. However, to facilitate optimum information transfer, not only the information itself and the sender should be taken into account, but also the receiver. To this end, attention should be paid to possible ways of increasing the level of health literacy in the population, especially across lower socio-economic strata. For instance, it has been suggested that more attention should be paid to health literacy competences in school curricula [40].

### Strengths and limitations

The health literacy measure that was used in this study differs from other measures, such as the Rapid Estimate of Adults Literacy in Medicine (REALM) and the Test of Functional Health Literacy in Adults (TOFHLA) [18,20]. These types of screening instruments largely measure health literacy in terms of reading skills applied in a clinical setting. The ability to read and understand health information is often referred to in the literature as ‘functional health literacy’ [2]. The HLS-EU-Q goes beyond measuring functional health literacy, as it focuses on multiple steps involved in information processing and decision-making in terms of health. It thereby provides an in-depth insight into health literacy as a multidimensional concept. Furthermore, the HLS-EU-Q aims to encapsulate a broad scope of health by extending across three domains as well as going beyond a merely patient-related context. A valuable addition for further research might be the further exploration of other factors such as motivation to perform the four health literacy competences. This could influence information processing and decision-making and therefore seems relevant for the identification of those at risk of perceived difficulties with health information [41,42].

An important strength of the methodology used in this study is that questionnaires were administered face-to-face. This facilitated the inclusion of adults with inadequate reading abilities. Adults who were not nationals of a European member state or had insufficient command of Dutch were excluded. Hence, the study does not reflect health literacy of adults with other ethnic backgrounds than European. To provide a rough indication of the proportion of the Dutch population that was not represented: in 2007, 8.3% of the Dutch population were born in a non-EU country [43]. It is likely that adults from ethnic minorities perceive more difficulties with health information, and hence the results might underestimate the health literacy skills of the adult population.

Another limitation is that elderly were overrepresented in the sample, which might have affected variables such as income or perceived social status and could possibly explain the small effect of income found in the current study, as the elderly account for a large proportion of the group of respondents with lower incomes. The educational level of older adults might have counterbalanced the effect of income on health literacy scores. In order to examine whether the findings of our study also yield for the subgroup of those aged 65 years or older, a subgroup analysis was performed, which indicated similar associations between socio-economic and demographic characteristics and health literacy (not tabulated). Therefore, the overrepresentation of elderly is not expected to have greatly affected the outcomes.

## Conclusions

Health literacy can be addressed as a concept that involves multiple dimensions that seem to differ in their perceived difficulty. The current study shows that health literacy varies for the competences of accessing, understanding, appraising and applying information across the domains of healthcare, disease prevention and health promotion. The findings are consistent with other research: those with a low socio-economic position, primarily with a low education level and a low subjective social status, have lower health literacy than those with a high socio-economic position.

## Consent

This study did not fall within the scope of the Netherlands Medical Research Involving Human Subjects Act and therefore did not require ethical approval. All potential respondents were contacted via telephone and informed about the aim of the study and the use of the data for analyses and publication. Participation was voluntary and potential respondents could opt out from the study at any moment. By participating respondents consented to the research procedure.

## Competing interests

The authors declare that they have no competing interests.

## Authors’ contributions

All authors made significant contributions to the conception of this study. IH analysed the data and drafted the manuscript with contributions of EU, JR, MD and KS. MS contributed to the data analysis and interpretation. All authors read and approved the final manuscript.

## Pre-publication history

The pre-publication history for this paper can be accessed here:

http://www.biomedcentral.com/1471-2458/13/179/prepub

## Supplementary Material

Additional file 1 Definition and integrated model of health literacy. Click here for file

Additional file 2 Characteristics missing values health literacy competences. Click here for file
