# Supplementary material for: Health literacy of Dutch adults: a cross sectional survey
Source: BMC Public Health. 2013 Feb 27;13:179. doi: 10.1186/1471-2458-13-179 (PMC3599856; doi:10.1186/1471-2458-13-179)
Supplement: Additional file 1 — Definition and integrated model of health literacy. [file 1471-2458-13-179-S1.docx]

**Additional file 1. Definition and integrated model of health literacy**

Based on a systematic literature review, a definition for health literacy was developed by the HLS-EU

consortium from which a conceptual model was derived.

**Definition** Health literacy is linked to literacy and entails people’s knowledge, motivation and competences to access, understand, appraise, and apply health information in order to make judgments and take decisions in everyday life concerning healthcare, disease prevention and health promotion to maintain or improve quality of life during the life course (Sørensen et al., 2012).

**Integrated model**


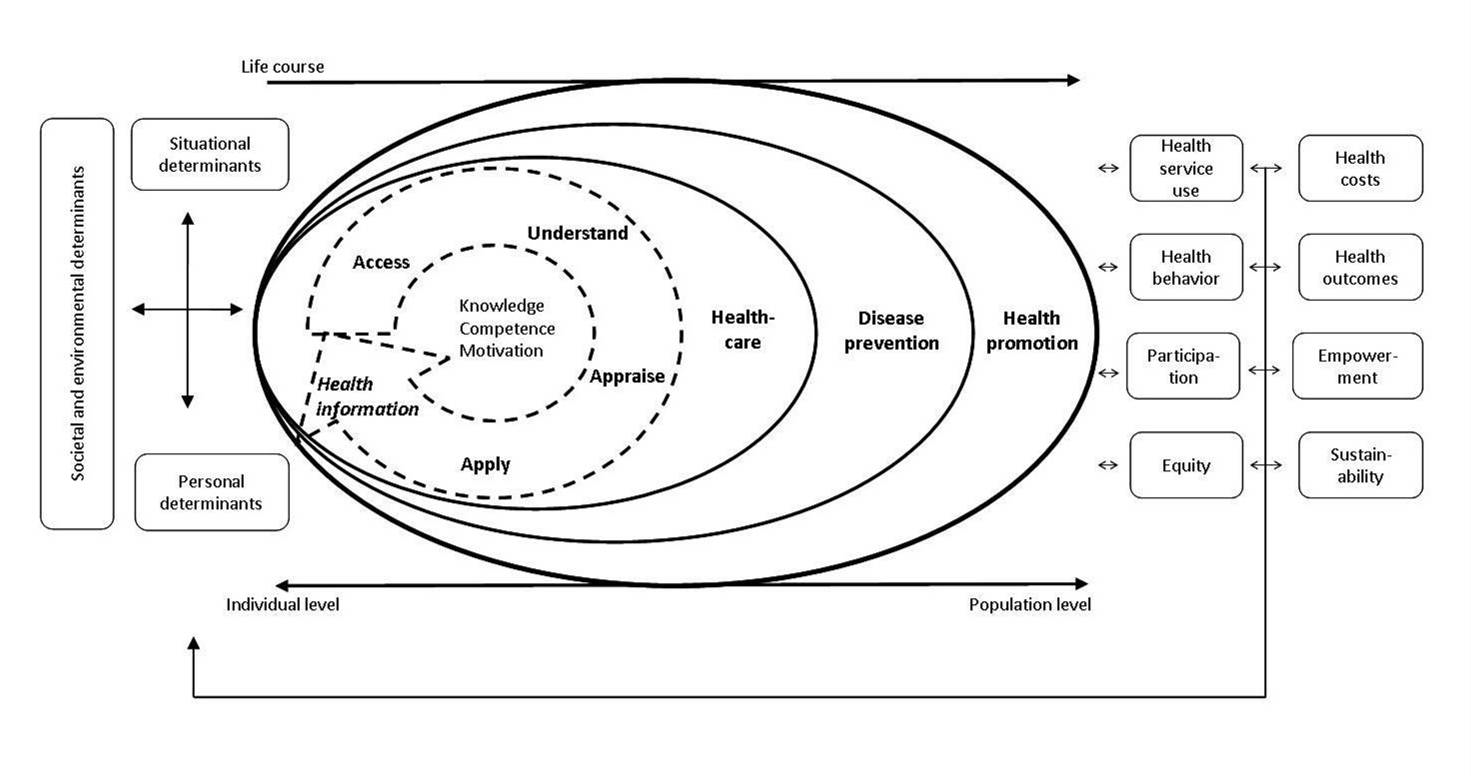


Sørensen, K et al.. 2012: Health literacy and public health: A systematic review and integration of definitions and models, BMC Public Health, 12(80).
