# Supplementary material for: Health literacy of Dutch adults: a cross sectional survey
Source: BMC Public Health. 2013 Feb 27;13:179. doi: 10.1186/1471-2458-13-179 (PMC3599856; doi:10.1186/1471-2458-13-179)
Supplement: Additional file 2 — Characteristics missing values health literacy competences. [file 1471-2458-13-179-S2.docx]

**Additional file 2. Characteristics of missing values on health literacy competences (%, N=440)**

|  | Respondents with one or more missing values on the health literacy items | | | | | |
| --- | --- | --- | --- | --- | --- | --- |
| Characteristics | Total  (n=440) | | Men  (n=221) | | Women  (n=219) | |
|  | % | n | % | n | % | n |
| Age in categories  25 – 34  35 – 44  45 – 54  55 – 64  65 – 74  75 – 84  85+ | 9.1  8.9  14.5  12.5  21.6  19.3  5 | (40)  (39)  (64)  (55)  (95)  (85)  (22) | 11.3  7.3  14.5  10.4  24.9  19.0  4.1 | (25)  (16)  (32)  (23)  (55)  (42)  (9) | 6.8  10.5  14.6  14.6  18.3  19.6  5.9 | (15)  (23)  (32)  (32)  (40)  (43)  (13) |
| Highest completed education level  No education or primary education  Lower secondary education  (Upper or post-) secondary non-tertiary education  Tertiary education (bachelor’s degree or higher)  Missing | 42.3  4.5  51.1  0.7  1.4 | (186)  (20)  (225)  (3)  (6) | 38.0  3.2  56.1  1.4  1.4 | (84)  (7)  (124)  (3)  (3) | 46.6  5.9  46.1  0  1.4 | (102)  (13)  (101)  (0)  (3) |
| Household net income per month in Euros  < 1850  1850 – 2400  2400 – 3600  3600 or >  Missing | 33.9  13.6  20.7  9.5  22.3 | (149)  (60)  (91)  (42)  (98) | 26.2  14.5  23.5  11.8  24.0 | (58)  (32)  (52)  (26)  (53) | 41.6  12.8  17.8  7.3  20.5 | (91)  (28)  (39)  (16)  (45) |
| Perceived social status  Low  Medium  High  Missing | 4.8  29.3  61.8  4.1 | (21)  (129)  (272)  (18) | 6.3  24.4  64.7  4.5 | (14)  (54)  (143)  (10) | 3.2  34.2  58.9  3.7 | (7)  (75)  (129)  (8) |
